# Psychosocial interventions for patients with chronic disease

**DOI:** 10.1186/1751-0759-6-2

**Published:** 2012-01-31

**Authors:** Hans-Christian Deter

**Affiliations:** 1 Medical clinic, Psychosomatics, Charité CBF, Hindenburgdamm 30, 12200 Berlin, Germany

**Keywords:** psycho social intervention, concepts, mechanism, efficiacy, eating disorders, pain, coronary artery disease

## Abstract

Treatment of patients with chronic diseases will be one of the main challenges of medicine in the future. This paper presents an overview of different origins, mechanism, and symptoms necessary for understanding new and different interventions that include a psychosomatic view.

In a psychosomatic therapeutic intervention there are very different targets, such as psychological symptoms, personality traits, attitudes toward disease and life, risk behaviour, and social isolation and as biological targets the change of autonomic imbalance and of the effects of the psycho-endocrinological or psycho-immunological stress responses. And there are also different psychosomatic measures that influence the individual biological, psychological and sociological targets. There is a need to give different answer to different questions in the field of psychosomatic and behavioral medicine. Comparative effectiveness research is an important strategy for solving some methodological issues. What is the target of treatment for different diseases: Symptom reduction, healing, or limiting progression to the worst case - the death of patients. We know that, the patient-physician relationship is important for every medical/therapeutic action for patients with chronic diseases.

This volume of BioPsychoSocial Medicine will present four different psychosomatic treatment studies from the clinical field in the sense of phase 2 studies: Reports of patients with obesity, anorexia nervosa, chronic somatoform pain and coronary artery disease were presented

## 

Bio psycho social medicine integrates different levels of research: The basic sciences focus on psychobiological mechanisms (e.g. psycho-neuroendocrinology, psycho-neuroimmunology, psycho-physiology) and psycho-somatic correlations. Various pathways have been formulated for different chronic diseases as have strategies for research on interactions between psychic phenomenon and biological functions found in different regions of the body, such as the brain-gut axis and the brain-heart axis. Medicine is not only a diagnostic discipline, but also a research field for medical practice - concentrating on physicians and other health care workers in action with their patients and focusing on the transfer of psychosomatic knowledge gained in the clinical practice of different populations and health care systems.

Treatment of patients with chronic diseases will be one of the main challenges of medicine in the future because they are very often influenced by psychosomatic or biopsychic factors. Additionally, the psychosomatic origins of many diseases are of interest and important to broader scientific discussion. In a historical view we know that, the patient-physician relationship [1] is especially important for every medical/therapeutic action for patients with chronic diseases. This also is supported by the new placebo literature that explains how the thinking, expectations, and trust of patients in the treatment procedures and in the person of the physician is essential for a positive response, e.g. reducing of symptoms or influencing the healing, of the disease [2,3]. This paper presents an overview of different origins, mechanism, and symptoms necessary for understanding new and different interventions that include a psychosomatic view.

Important stressors and risk factors for developing a chronic disease, worsening it, or increasing their chronicity include pathological mechanisms involved in the ethiopathogenesis of disease, psychological mediators, behavioural risk factors, the severity of the disease itself, and their somatic, psychological and sociological effects (Table 1).

**Table 1:** Bio psycho social origins of chronic diseases (e.g coronary heart disease, bronchial asthma, rheumatoid arthritis, inflammatory bowel disease, essential hypertension, atopic dermatitis, diabetes, cancer and others; modified after Orth-Gomér et al 1997)

Origins of Disease	Factor 1	Factor 2	Factor 3	Factor 4	Factor 5	Factor 6	Factor 7
Stressors	work stress	family stress	social competition	sensitive kind of aware ness	inner psychic conflicts		
Psychological mediators	depression	anxiety	hostility	denial	type A	type C	type D

Behavioral risk factors	smoking	sedentary life style	poor nutrition	alcohol consumption	suicidal behavior	no care for the own person	over active

Pathological mechanism	genetic	autonomic im-balance	psycho-immunological path-ways	psycho-endocrinological path-ways	inflammation	lipids/ glucose	coagulation

Severity of disease							

somatic	bodily complaints	bodily limitations	pain				

psychological	psychic symptoms	chronic depression	suicidal tenden-cies	chronic anxiety	too many doctor visits	too many drugs	social inhibition

sociological	low social functioning	social isolation	unable to work	low income	high treatment costs		

Thus, in a psychosomatic therapeutic intervention there are very different targets, such as psychological symptoms, personality traits, attitudes toward disease and life, risk behaviour, and social isolation and as biological targets the change of autonomic imbalance and of the effects of the psycho-endocrinological or psycho-immunological stress responses (Table 2).

**Table 2:** Different psychological, biological and sociological targets forchronic diseases

Targets of psychosomatic therapy	Target 1	Target 2	Target 3	Target 4	Target 5
**Psychological** reducing symptoms	depression	anxiety	hostility	pain	

changing personality traits	self esteem	salutogenesis	type A	type C	type D

changing attitudes to disease and life	coping abilities				

**Behavioral** changing behavior	adaption of illness behavior	smoking, alcohol	eating habits Vegetables and Low fat	physical activity	

**Sociological** Gaining social support	social activities	going to work	better relationship with part ner/spouse	better relationship to other family members	better relation ship to friends a. neighbours

**Biological** **targets**	harmonize the autonomic imbalance	influence the psycho- endocrino- logical stress response	influence the psychoen- docrinological stress response -cortisol, catecholamine axis)		
	influence pathogenic psychoin- flammatory mechanism	influence pathogenic psycho- immunologic mechanism			
	influence cerebral regulation of the stress response				

**Biological** **Targets:** **Disease** **oriented** **measurements**	asthma: reduce tendencies for attacks of hyperventi lation	diabetes: reduce phases of hyper and hypo glycemia	atopic dermatitis: reduce phases of cratching	essential hy- Pretension: handel hypertensive crisis after emotional activation	

**Disease orient-** **ted management**	information about disease	make regulatory doctor visits	behaviour according disease management needs	take regulatory drugs	

However, there are also different psychosomatic measures that influence the individual biological, psychological and sociological targets (Table 3). To date, there are no convincing studies that demonstrate which treatment regimen is helpful for which target in which chronic disease. There are many reports of experiences in the clinical field related to psychosomatic therapeutic support for chronic diseases [4], but in this field the main work lays in front of us. Methodological questions have arisen as to which direction we should take to make the next steps of research. Linda Powell and coworkers [5] gave interesting advice here, comparing the situation with the pharmacological field: Phase 1, phase 2, and phase 3 studies are all helpful and necessary for different tasks in related to this situation of medicine (Table 4). There is a need to give different answer to different questions in the field of psychosomatic and behavioral medicine.

**Table 3:** Different psychological, biological and sociological therapeutic interventions for chronic diseases by general practitioners, medical specialists and psychotherapists

Psychosoma- tic therapy	**Individual**, group insight **therapy**,	**Individual**, group CBT	Relaxa- tion	**Family**, social therapy	Mentali- sation	Psycho- pharma ceuticals	Other drug therapy
**Psychological** Reducing of symptoms	+++	+++	++	+		++	+

Changing personality traits	+++	++					

Changing atti- tudes to di- sease and life	++	++					

**Behavioral** changing behavior	+	+++	physical training groups	+			+

**Sociological** Gaining social support	++	++		+++			

**Biological** **targets** Harmonize the autonomic imbalance	+	+	+++				

Influence pathogenic psycho-inflam- matory mechanism	+?	+?	++?	?	?	?	?

Influence cerebral re- gulation of the stress response	?	?	?	?	++?	?	?

Disease oriented measurements	+	++	++			+	+++

Disease orient- ted management							+++

**Table 4:** Psychosomatic/behavioural research strategies for treatment trials in chronic diseases (according to L. Powell (2010)

Activities	Targets
1. Identify	mechanism and pacemaker of chronic diseases

2. Evaluate	effects of different psychosomatic therapeutic interventions in chronic diseases in a regular clinical setting (phase 1 and phase 2 studies)

3. Evaluate	therapeutic effects in different diseases in randomized controlled trials (RCT, phase 3 studies)

4. Bring back	the forthcomings of RCT's in the clinical field. Make observational studies for all unselected patients

5. Specify	different targets of bio psycho social medicine in chronic diseases and make again phase 1 and phase 2 psychosocial intervention studies for it

6. Compare	different therapeutic strategies/interventions for different targets in one chronic disease

This volume of BioPsychoSocial Medicine will present different studies from the clinical field in the sense of phase 2 studies. Trials of representative chronic diseases using mostly unselected patient samples and special issues, but no randomized controlled trials (RCT) will be reported. Although scientific, clinical, and social medical discussion is important, phase 3 studies give proof of only one very specific and limited treatment effect in one patient group, which does not accurately present the clinical reality and complexity. The floor is open for more phase 1 and phase 2 studies:

1. Some authors focus their intervention on only one important factor - on worrying as a predictor for the development of cardiovascular diseases, such as essential hypertension [6]. This study is an example for focusing a psycho social intervention on a single psychic variable argued as responsible for the development of disease. Here the technique of the behavioral intervention seems very simple but successful in targeting the important influencing factor, such as depression in studies with coronary artery disease [7].

2. Another important aspect is the kind and dosage of drugs used in a psychosomatic therapy. Is it sufficient to treat depression or anxiety in a chronic disease with psychopharmaceuticals alone, or it is necessary to apply additional psychosocial interventions. If so, what amount of psychotherapy is helpful: 10 minutes more conversation with the physician compared to therapy as usual (TAU), or one, two or fifteen hours more than TAU? Moreover, which psychotherapeutic strategy is necessary: relaxation, cognitive, behavioral, or insight therapy, and in which therapeutic setting: individual therapy, group therapy, or a combined therapeutic program in an outpatient or inpatient setting? The basis for all these interactions is a trustful relationship between the patient and therapist, which means that the therapist has to acknowledge the needs and low self esteem of patients and to give hope and trust in the future through professional activity and good relations with the family and friends, all with the intention of minimizing symptoms. Kristina Orth-Gomér showed the usefulness of these activities in their gender segregated groups. Men and women with coronary heart disease seem to do better if they are kept apart in psychotherapeutic groups. It is very impressive to see the way in which such a program of intensive psycho social intervention can influence the psychic and somatic condition of patients.

3. Therapeutic activity depends not only on the developed health care situation of an individual country, such as Japan, Germany or the US, but also on the research results on special diseases worldwide. Standardization has been done in evidence based medicine (EBM) according psychosocial interventions for different diseases. Great differences in international guideline can be found, i.e. in anorexia nervosa therapeutic measurements between the NICE guidelines of the UK and the German S3 guidelines for eating disorders [8,9].

3. What is the target of treatment for different diseases: Symptom reduction, healing, or limiting progression to the worst case - the death of patients with anorexia nervosa [10]. After a complex, high intensity inpatient therapy, it is important to analyse the conditions that foster a bad outcome and determine the psychosocial factors and psychotherapeutic interventions that improve the prospects for a better long term outcome. In this issue, Laurence Erdur et al present predictors for a dead end after intensive inpatient psychotherapy of Anorexia nervosa.

On this point we have to acknowledge the level of evidence based medicine and the importance of randomized controlled trials (RCT) for findings in psychosomatic medicine - the only argument for what counts in the discussion of the somatic disciplines [11]. But, we also have also to differentiate between "phase 2" and "phase 3" studies in a pharmacological sense [4]. In this situation, we need many phase 2 studies to show the effectiveness and not only the efficacy of a targeted psychosocial intervention in a special situation for a special patient group. Comparative effectiveness research is an important strategy for solving these methodological issues [12].

This seems also true in the complex field of patients with pain disorders. Bernd Bergander and collegues show in their article the situation the conditions and the success of a complex inpatient treatment program for these patients. They demonstrated that there are differences between older and younger patients in the concept and outcome of treatment. Besides a good relationship between physician and patient, there is a need to understand the individual condition of patients, but it is also an advantage if the physician has good experience in treating this kind of patients over a long period of time and in a somatic and psychological way.

From psycho analytic studies we know that the attachment style of an individual - developed since childhood- is important to the success of a good patient relationship: Sybille Kiesewetter et al showed in their study of patients with obesity the importance of this factor and the helping alliance between patient and therapist in a weight loss program designed to promote a positive change of the body mass index of these patients.

Who is responsible for psychosocial intervention: The physician, the internist, the psychotherapist, the psychologist, the nurse, or the social worker? There is no doubt that every person involved in a psycho social intervention should be well versed in the kind of somatic/pharmacological therapy for this disease. He/she should additionally have good communicative ability, a cooperative attachment style, and the capability of understanding patients as biographical, psychological, and sociological beings. This seems to me a non-negligable basis of a psycho social intervention for chronic disease patients. Additionally, the therapist needs knowledge about the risk factors that influence the beginning, course, and end stages of disease as well as the ability to change the thinking and behavior of the patient.

One question remains: All four studies in this volume are without a control group, e.g. phase 2 studies. They show efficiacy in a clinical setting without special selection of patients using standardized measurements and methods. Naturally, there is a need for additional randomized controlled trials, but to date the medical field is very heterogenous in regard to all diseases that are influenced by psycho social factors. Gender, age, severity, and duration of disease and the severity of psychiatric co-morbidity as well as drug intake are additional factors that influence the disease condition and the kind of psychosomatic somato/psychic mechanism. The field is open for different psycho social interventions on different levels for different diseases, controlling for all the factors mentioned above [13]. As a first step, we must show the effectiveness of such interventions for several diseases - epidemiological and socio medically important and influenced by psychosomatic factors. If we achieve good and impressive results, it will make it possible to change the medical practice in Japan, and all over the world [14]. This volume of BPSMedicine presents four studies to show examples of the ways we can to go in preparing and conducting psycho social medical interventions..
